# Supplementary material for: COVID-19 vaccine acceptance and hesitancy in N’Djamena, Chad: A cross-sectional study of patients, community members, and healthcare workers
Source: PLOS Glob Public Health. 2022 Jun 27;2(6):e0000608. doi: 10.1371/journal.pgph.0000608 (PMC10022375; doi:10.1371/journal.pgph.0000608)
Supplement: S1 Text — (DOCX) [file pgph.0000608.s002.docx]

SSA Vaccine Hesitancy Survey

Initials of participant

________________________________________________________________

Date of enrollment in 2021 (dd/mm)

________________________________________________________________

Location of enrollment

- Hospital inpatient
- Hospital outpatient
- Community setting (please specify) ________________________________________________
- Other (please specify) ________________________________________________

1. Have you received the COVID-19 vaccine? (Circle one)

- Yes
- No
- Don't know

1b. Which COVID-19 vaccine did you receive?

- Pfizer-BioNTech
- Moderna
- AstraZeneca
- Johnson & Johnson
- Novavax
- Sinopharm
- Don't know

2. If the COVID-19 vaccine were generally available, would you take the vaccine?

- Yes
- No
- Don't know

3. Please state how much you agree with each of the following statements by choosing a number below on a scale from 1 (strongly disagree) to 5 (strongly agree).

|  | 1 (strongly disagree) | 2 | 3 (neither agree nor disagree) | 4 | 5 (strongly agree) |
| --- | --- | --- | --- | --- | --- |
| COVID-19 vaccination is important for my health |  |  |  |  |  |
| COVID-19 vaccines are effective |  |  |  |  |  |
| Having myself vaccinated for COVID-19 is important for the health of others in my community |  |  |  |  |  |
| The COVID-19 vaccines offered by the government program in my community are beneficial |  |  |  |  |  |
| COVID-19 vaccines carry more risks than older vaccines |  |  |  |  |  |
| The information I receive about COVID-19 vaccines from the vaccine program is reliable and trustworthy |  |  |  |  |  |
| Getting COVID-19 vaccines is a good way to protect myself from disease |  |  |  |  |  |
| Generally, I do what my doctor or health care provider recommends about vaccines |  |  |  |  |  |
| I am concerned about serious adverse effects of the COVID-19 vaccine |  |  |  |  |  |
| I do not need vaccinated for COVID-19 because it does not present a risk to me |  |  |  |  |  |

4. Please choose either Yes or No based on your responses to the following questions.

|  | Yes | No |
| --- | --- | --- |
| Do you believe that a vaccine can protect you from the COVID-19 disease? |  |  |
| Do you think that most people like you will take the COVID-19 vaccine? |  |  |
| Have you ever been reluctant or hesitant to get a vaccination? |  |  |
| Have you ever refused a vaccination? |  |  |
| Has distance, timing of clinic, time needed to get to clinic or wait at clinic and/or costs in getting to clinic prevented you from getting a vaccine? |  |  |
| Are there other pressures in your life that prevent you from getting the COVID-19 vaccine? |  |  |
| Are there any reasons you think you should not be vaccinated for COVID-19? |  |  |
| Do you think that it is difficult for some ethnic or religious groups in your community to get COVID-19 vaccinations? |  |  |
| Have you ever received or heard negative information about the COVID-19 vaccination? |  |  |
| If you have heard negative information about the COVID-19 vaccine, would you still get vaccinated after you heard the negative information? |  |  |

5. Please circle either Yes or No based on your responses to the following questions.

|  | Yes | No |
| --- | --- | --- |
| Do religious leaders in your community support COVID-19 vaccination? |  |  |
| Do political leaders in your community support COVID-19 vaccination? |  |  |
| Do teachers in your community support COVID-19 vaccination? |  |  |
| Do healthcare workers in your community support COVID-19 vaccination? |  |  |

6. Where do you get information about the COVID-19 vaccine? (Select all that apply)

- Television
- Radio
- Social media
- Local organizations
- International organizations (such as World Health Organization)
- Healthcare provider
- Traditional healer
- Friends
- Family
- Co-workers
- Other: ________________________________________________
- None of the above

7. What concerns would you have about taking the COVID-19 vaccine? (Select all that apply)

- No concerns
- Whether it would be effective
- Whether it would be safe
- Religious concerns
- Fear of needles
- I do not think COVID-19 presents a risk to me
- I had a bad experience with a previous vaccine
- Concerns about the pharmaceutical industry
- I don’t trust the government
- Concerns from TV/radio/news
- Concerns from social media
- Concerns about transit
- Concerns about cost
- Concerns about side effects
- Prefer to use traditional healing practices

8. Please specify any other concerns you have about receiving a COVID-19 vaccine:

_______________________________________________________________

9. Have you experienced any symptoms associated with COVID-19 in the past year? (Select all that apply)

- Fever
- Tiredness
- Dry cough
- Aches and pains
- Nasal congestion (stuffy nose)
- Loss of smell
- Loss of taste
- Headache
- Runny nose
- Shortness of breath
- Sore throat
- Pneumonia
- Diarrhea
- None of the above

10. Have you ever been tested for COVID-19?

- Yes
- No
- Don't know

10b. What was the test result? (Circle all that apply if you have been tested more than once.)

- Positive
- Negative
- Could not be determined
- Don't know

11. Do you personally know anyone who had COVID-19?

- Yes
- No
- Don't know

12. Do you personally know anyone who was hospitalized because of COVID-19?

- Yes
- No
- Don't know

13. Do you personally know anyone who has died because of COVID-19?

- Yes
- No
- Don't know

14. Age (in years):

________________________________________________________________

15. Sex (circle one):

- Male
- Female
- Another identity: ________________________________________________

16. Race (circle one):

- Black
- Another identity: ________________________________________________

17. Which of the following best describes you? (circle all that apply)

- Patient
- Family member of a patient
- Healthcare worker
- Traditional healer
- Other: ________________________________________________

18. Highest education level completed:

- no school
- primary school
- secondary school
- high school
- university

19. What is your employment status?

- Employed full-time
- Employed part-time
- Unemployed
- Retired
- Student
- Other: ________________________________________________

20. Main occupation:

________________________________________________________________

21. Religion:

- Muslim
- Christian
- Animist
- Other: ________________________________________________

22. Do you have a previously known medical history of any of the following? (circle all that apply)

- Depression
- Diabetes Mellitus
- Alcohol use
- Tobacco use
- Drug use
- Current pregnancy
- Stroke
- Hypertension
- Headache or migraine
- Seizures/epilepsy
- Obesity
- HIV
- Kidney disease
- Heart disease
- Liver disease
- Cancer
- Chronic lung disease

23. Please circle either Yes or No based on whether your household owns each of the following items:

|  | Yes | No |
| --- | --- | --- |
| Electricity |  |  |
| Radio |  |  |
| Refrigerator |  |  |
| Television |  |  |
| Non-mobile telephone |  |  |
| Mobile telephone |  |  |
| Bicycle |  |  |
| Motorbike/scooter |  |  |
| Car |  |  |
| Truck |  |  |

24. How many people live with you in your household?

________________________________________________________________

25. How much money has your household earned in the past month (in your local currency)?

________________________________________________________________

[if participant was a patient, survey administrator inputs diagnosis here]:

________________________________________________________________
