# Supplementary material for: COVID-19 vaccine acceptance and hesitancy in N’Djamena, Chad: A cross-sectional study of patients, community members, and healthcare workers
Source: PLOS Glob Public Health. 2022 Jun 27;2(6):e0000608. doi: 10.1371/journal.pgph.0000608 (PMC10022375; doi:10.1371/journal.pgph.0000608)
Supplement: Attachment — Submitted filename: Chad_VH_PLOSGPH_RtoR_220418.docx [file pgph.0000608.s005.docx]

18 April 2022

Dear editorial team of *PLOS Global Public Health*,

We appreciate the thoughtful feedback and thank you for your time and consideration. Please find below a point-by-point explanation of revisions to our manuscript, *“COVID-19 vaccine acceptance and hesitancy in N’Djamena Chad: Results from a cross-sectional study of patients, community members, and healthcare workers,”* in response to reviewer feedback.

Comments from Reviewer 1:

In the submitted manuscript titled: COVID-19 vaccine acceptance and hesitancy in Chad: A cross-sectional study of patients, community members, and healthcare workers”, the authors described the situation of COVID-19 vaccine uptake among different groups in Chad. Below are points that need to be clarified:

1. Abstract: Please include the main aim of this study in the introduction section of the abstract.

We have added the following sentence to the introduction section of the abstract: “*We aimed to study COVID-19 vaccine acceptance and hesitancy among community members, patients, and health care workers in urban Chad.”*

2. Introduction: a. First paragraph: You may wish to give a global picture of COVID-19 vaccine uptake and hesitancy, then narrow down

Thank you for the suggestion. We have reorganized the paragraphs of the introduction to now present the global view of COVID-19 vaccine hesitancy (previously paragraph 3 of introduction) before narrowing down to our target population in Chad.

3. b. “Since the beginning of Chad’s vaccination campaign, people over 65 years of age, medical staff, and candidates for the Hajj pilgrimage have been prioritized for immunization, although the Chadian Health Minister has stated that “vaccination is open to all.” This statement is contradictory, kindly rephrase to fit your manuscript

We have rephrased this sentence. It now reads, “*The Chadian Health Minister has stated that “vaccination is open to all,” and people over 65 years of age, medical staff, and candidates for the Hajj pilgrimage have been prioritized for immunization since the beginning of the vaccination campaign.”*

4. c. “From the beginning of its vaccination campaign to 12 December 2021, Chad has administered 285,922 COVID-19 vaccine doses—sufficient to fully vaccinate less than one percent of the Chadian population” This sentence may be moved to the above paragraph as it discusses similar concepts as in para 1, the remaining sentences can be merged with para 3

In line with our response to Comment #2, we have moved this sentence to fit more appropriately with the paragraph discussing Chad-specific COVID-19 information. The remaining sentences have been merged, and our introduction now features only three paragraphs.

5. d. “One factor at the individual level preventing COVID-19 vaccine uptake is vaccine hesitance,” This statement sounds controversial, as vaccine hesitancy is a broad term by itself. Please clarify this statement in relation to the reference cited

We have rephrased this sentence, which is now in the opening paragraph of the introduction. It now reads*, “A self-reported factor associated with vaccine uptake is vaccine hesitancy, defined by the World Health Organization as the . . .”*

6. e. Please reduce the number of preprints cited as there are a lot of published papers in covid-19 vaccine acceptance and hesitancy

Where possible, we have updated the preprints which are now published. Unfortunately, there is a paucity of full publications regarding the specific issues we present in this paper and we were not able to remove all preprints. Where applicable, we have identified citations in the text as pre-prints for transparency (e.g., “*In a* ***preprint*** *of a review of COVID-19 vaccine hesitancy in 11 countries in the Sub-Saharan African (SSA) region, …”).*

7. f. “a country with a very young population and with lower average per capita wealth than many other Sub-Saharan African countries” Maybe not necessary to be added in this specific section

Removed.

8. g. “In this study, we aim to characterize vaccine acceptance and hesitancy in Chad and report on demographic, clinical, and socioeconomic associations “ A brief review of literature on these factors may be added

Thank you for the suggestion. Although we had no pre-specified hypotheses about these associations, we have added some statements on our motivation. *There is research from Ghana suggesting that COVID-19 vaccine hesitancy may be associated with various sociodemographic factors such as age, gender, and outlet of primary information receipt.^ref^ Other work finds no differences in COVID-19 vaccine hesitancy among six countries in SSA by demographic factors, but does suggest differences by socioeconomic factors, such as education level and household wealth.^ref^ No research to our knowledge has looked at possible associations of COVID-19 vaccine hesitancy in Chad.”*

9. h. Please include your aim of conducting this study towards the end of the introduction section

At the end of the introduction, we now state “*In this study, we aim to characterize vaccine acceptance and hesitancy in N’Djamena, Chad and report on demographic, clinical, and socioeconomic associations with acceptance and hesitancy in patients, community members, and healthcare workers.”*

10. i. In the introduction I have failed to capture the reason of the choice of the study population in this research. Please include a paragraph explaining why you chose health care workers, patients and community members. How are these populations linked? Any rationale of such grouping? Please back up using literature in each specific group

*We have added a paragraph to the introduction addressing our reasoning for the selected groups, with appropriate references (Paragraph 3 of Introduction). Specifically, we state “Some emerging research suggests that healthcare workers in the African region (e.g., Ethiopia) endorse significantly high levels of vaccine hesitancy (ref), even though it may be expected that healthcare workers are less likely to be vaccine hesitant than the general population. Some research from Zambia suggests level of personal COVID-19 vaccine hesitancy may be associated with beliefs about personal risk of infection (ref); this suggests that patients in hospitals, who tend to have more comorbidities and compromised immune systems than others, may also have different levels of vaccine hesitancy compared to the general population. For these reasons, we decided to conduct our study aims amongst three main groups: healthcare workers, patients, and general community members.”*

11. Methods. a. Kindly check the journal style if ethics approval comes first in the section

After checking the journal style guidelines, we note that our presentation of the ethics approval section is appropriate. Thank you.

12. b. Please include ethical approval numbers for each institution

We have added the protocol number assigned by the Mass General Brigham site as appropriate. The Chadian site simply refers to this protocol by its title, but we are happy to submit supporting documentation on the approval if required.

13. c. “Participants’ independent completion of the survey was considered to imply consent to participate in the study” this statement is not clear, kindly clarify

We have removed this confusing language and now more clearly state *"Informed consent was obtained verbally by study staff from each participant in their preferred language prior to survey commencement, and survey initiation implied consent to participate. Separate written consent forms were not required.”*

14. d. Setting: Kindly align your study setting section to fit the actual context of the study according to your study population and objective of your study

We have adjusted the content of this section to now be relevant to N’Djamena given the context and objective of our study: “N’Djamena is the capital city of Chad and contains roughly 10% of the Chadian populace (2010 population of approximately 1.8 million).^ref^ N’Djamena is home to one of the two teaching hospitals in Chad—the University Hospital Center of National Reference.”

15. e. Please include study design and duration

We have added a section on these topics to the methods, which details the following: “This cross-sectional study was conducted from August to October 2021 using a one-time participant survey, administered by study staff.”

16. f. Recruitment and enrollment: How different was the recruitment process between the three different target groups; community members, HCWs and patients? Briefly expand according to your study population

We have added three sentences to this effect to the “recruitment and enrollment” section. We now state: *“All participants in the patient group were recruited at the National Reference Hospital, N’Djamena. Participants in the community members group were recruited from public spaces, such as marketplaces, in the greater N’Djamena area. Participants in the healthcare providers group were recruited from both the National Reference Hospital and public spaces in greater N’Djamena.”*

17. g. Materials: Needs to be specified, Self-administered questionnaire, interviewing forms, online tool? Was it the same method used in each study population?

We already state clearly in the materials section the following: *“Study staff, who were Chadian medical students (AB, ART, AG, and DF), administered the 25-question, 15-minute survey in-person to participants and recorded responses either on paper or electronically via Qualtrics^TM^.” The survey instrument is also available as an appendix.*

18. h. In the materials you mentioned knowledge: Please clarify in the aim of this study if knowledge was also part of it, if yes, please add literature on knowledge as well. Note that since you have different groups, the method to assess knowledge cannot be uniform across

The reviewer makes an important point: We did not assess *knowledge* of these groups regarding COVID-19 and have updated the manuscript where appropriate to discuss “attitudes and practices” regarding COVID-19.

19. i. Participants and sample: This sample size needs to be justified-what was the basis of 500? You need to justify your sample size and explain how this convenience sampling was done. If your sample size is justified and adequately powered you may wish to stratify your regression analysis by study population

As mentioned in the text, our sample size was determined based on feasibility of conducting the study. We have tried to increase transparency in this method and now state: “*For this observational study, we set an arbitrary target of 500 participants based on available time and resources.”* As mentioned in the text, the convenience sampling was done by approaching patients within the hospital and asking them to participate (although we were unable to collect a response rate), by approaching community members at public spaces in the city (e.g., marketplaces), and, as we have now clarified, by approaching *“healthcare providers from both the National Reference Hospital and public spaces in greater N’Djamena.”* Because we did not calculate a sample size *a priori* and because our final regression model shows no significant differences between the three groups regarding vaccine hesitancy, we have not stratified the regression analyses.

20. j. Outcome and data analysis “ WHO SAGE Vaccine Hesitancy Survey (see supplementary materials)” should be mentioned in study instruments/materials. Also explanations on each specific question is not necessary. It is also safer to use the term open ended question rather than “qualitative aspect”

We have added the WHO SAGE VHS to the study instruments/materials section. We have reduced the discussion on specific items in this measure also. Finally, we have changed all references to “qualitative aspect” or “qualitative measure” throughout the survey to “open-ended measure.”

21. Results: a. “We enrolled 516,….508….. however in methods its 500, please be consistent

The numbers presented accurately reflect the conduct of this study. As mentioned in the methods, we set a target sample size of 500 participants. As mentioned in the results, we actually enrolled 516 participants (multiple staff members independently enrolled participants throughout the workday and we were unable to stop enrollment exactly at 500 on the last day of data collection). We state that 8 patients had incomplete data, so a final sample of 508 participants’ data was analyzed for this manuscript.

22. b. You don’t have to repeat every item found on the table in the text. Please include important items only

Thank you for the suggestion. We have significantly reduced the table-text redundancy throughout the results section.

23. c. “When asked if they knew anyone who had ever been hospitalized for COVID-19, 43% responded affirmatively (of them, 45% were healthcare workers); approximately one-third responded that they knew someone who had died due to COVID-19 (of them, 39% were healthcare workers)” . It may not be easy to explain this if healthcare workers, community members and patients are considered under one basket. These groups are different

We agree that these are distinct groups and should not be considered under one basket. Data for these items are already present in the tables, broken down by each of the three groups, so we have elected to delete this paragraph to reduece redundancy.

24. Discussion: a. In this work, we establish an evidence base for work on COVID-19 vaccine acceptance and hesitancy in Chad through a mixed methods study of patients, community members “ I think this is not a mixed methods study, it is simply a quantitative study with open ended questions

We have deleted “mixed-methods” in the discussion.

25. b. Please clarify the study population in reference 16 and 17

Reference 16 discusses an article published by the Africa CDC looking broadly at 15 different countries across SSA. This is clarified twice in-text, e.g., “A previous study of 15 countries in SSA conducted by the Africa CDC found a significant association between more vaccine hesitancy and social media use.^16^” Reference 17 discusses vaccine hesitancy among healthcare workers across SSA and we clarify this population with the following: “vaccine hesitancy among healthcare workers in Zimbabwe, Ghana, South Africa, Kenya, Sudan, and Ethiopia.^17^”

26. c. One of the strengths mentioned is large sample size, please explain in the method section how this study is powered considering all the three groups. Or modify the statement accordingly

Thank you for the suggestion. We have added the following sentence to the methods section: *“Although we did not complete an a priori power calculation, based on an estimated 166 people per study group, there would be 80% power to detect at least a 13-point difference in two proportions with a level of significance of 0.05.”*

Comments from Reviewer 2:

1. This study contributes to the growing body of knowledge in preparation for full COVID-19 campaigns to improve coverage. it highlights key information that need to be addressed through communication to ensure that COVID-19 AND vaccination are understood by the community. However, the tittle says " COVID-19 vaccine acceptance and hesitancy in Tchad. However, they sampled only one reference hospital. Convenience sampling is OK, but to reflect Tchad as a whole, it will be necessary to sample at least a few other hospitals. Moreover, the number of community members was few to represent Tchad. Sampling more patients in other hospitals and participants of other sector of the community will be more representative.

Thank you for pointing out this issue, which we also address in our limitations, *“Because this was a convenience sample in an urban area, we are unable to make population-based assumptions about vaccine hesitancy in Chad nationally.”* We have further addressed this limitation by amending the title to ensure it does not imply our paper represents Chad as a whole. The new title more accurately reflects “*COVID-19 vaccine acceptance and hesitancy in* ***N'Djamena, Chad****: Results from a cross-sectional study of patients, community members, and healthcare workers*.”

Comments from Reviewer 3:

This is a great piece on vaccine acceptance and hesitancy in Chad and associated demographic, clinical, and socioeconomic associations. The authors started with a helpful introduction on the COVID-19 vaccination efforts and challenges in Chad thus far and a rationale as to why it is important to understand vaccine acceptance and hesitancy in order to inform public health campaigns. They also provide a useful background on the Chadian setting and the country’s demographics and existing health and wellness challenges. They then go on to explain how they conducted their survey and collected responses; and then on to highlight the results from their survey and analyses. While there are some limitations to their methods, the authors clearly acknowledge these and state the rationale for conducting the study in the way that they did. They also discuss how these limitations (e.g. selection bias) may have impacted results and in what direction. Overall, the methods appear to strong despite these issues. Authors stated that anonymous data could be made available on request. I found the results and discussion to be very well written with the right amount of word space designated to the findings. I found the lack of a statistically significant association between vaccine hesitancy and social media use to be very interesting. Might this have anything to do with the higher levels of education among study participants? I thought the piece was already very good, and did not have any suggestions for significant changes/improvements (a few very minor edits are listed below). The article is well-written and clearly presented. It will be a great contribution to the literature base as the first account of COVID-19 vaccine acceptance and hesitancy in Chad. Thank you for the opportunity to review this.

Thank you for the thoughtful review of our manuscript. We did not study educational level beyond the health educated group vs. community. We do however show the educational level of our respondents in the tables and mention in our discussion the following: *“An important characteristic of our study population is the high number of participants who had at least a high school education (nearly 80%) which is much higher than the proportion in Chad as a nation. This made variations among people with less than a high school education too few to fully analyze in this study.”*

Minor suggestions:

1. First paragraph of introduction – I suggest rephrasing to something more along the lines of: Because of testing and reporting issues – particularly early in the pandemic – it is likely that the true number of positive cases in the country is larger than the official count.

We have amended this sentence to read: “*It is possible the true number of cases is larger than the official count, due to issues of testing and reporting, especially early in the pandemic.”*

2. Page 2 – “However, there have been no reports” – would suggest adding “To our knowledge” somewhere in this statement

We have amended this sentence to read: “*However, to our knowledge there have been no reports of COVID-19 vaccine impressions and beliefs to date from Chad, ...”*

3. Page 11 – might be useful to define anosmia and ageusia in non-technical terms in parentheses

We have amended these fragments to: “*anosmia (loss of smell)”* and *“ageusia (loss of taste).”*

4. Page 19, final para – would adjust sentences to “A previous study conducted by the Africa CDC of 15 countries in SSA found XXXXX. While more than two-thirds of participants in our study reported using social media as an avenue for seeking COVID-19 information, we did not find a significant association between higher levels of vaccine hesitancy and higher use of social media, where vaccine mis- and dis-information are likely to prevail.”

We have amended these sentences to read: “*A previous study of 15 countries in SSA conducted by the Africa CDC found a significant association between more vaccine hesitancy and social media use.^16^ While more than two-thirds of participants in our study reported seeking COVID-19 information through social media use, we did not find a significant association between higher levels of vaccine hesitancy and use of social media, where vaccine mis- and dis-information are likely to prevail.”*

5. In your final paragraph, I suggest adding reference(s) to some of the other barriers of vaccination uptake when you mention this.

We have added a brief list of various “other barriers” to vaccine uptake and have included relevant references.

Specifically, we now state: *“Although many barriers exist to this pace of vaccination uptake in SSA (such as vaccine access,^24^ community engagement,^25^ travel to vaccination sites,^26^ etc.), addressing the issues of vaccine hesitancy reported here presents one avenue through which vaccine uptake may be bolstered.”*

---

Thank you again for the constructive comments and detailed reviews.
