# Supplementary material for: COVID-19 vaccine acceptance and hesitancy in N’Djamena, Chad: A cross-sectional study of patients, community members, and healthcare workers
Source: PLOS Glob Public Health. 2022 Jun 27;2(6):e0000608. doi: 10.1371/journal.pgph.0000608 (PMC10022375; doi:10.1371/journal.pgph.0000608)
Supplement: Attachment — Submitted filename: Chad_VH_PLOSGPH_RtoR_220511.docx [file pgph.0000608.s006.docx]

11 May 2022

Dear editorial team of *PLOS Global Public Health*,

We again appreciate the thoughtful feedback and thank you for your time and consideration. Please find below a point-by-point explanation of revisions to our manuscript, *“COVID-19 vaccine acceptance and hesitancy in N’Djamena Chad: Results from a cross-sectional study of patients, community members, and healthcare workers,”* in response to reviewer feedback.

Comments from Reviewer 1:

None

Comments from Reviewer 2:

None

Comments from Reviewer 3:

1. Thanks for the opportunity to re-review the piece and thank you for considering and addressing the previous reviewer comments. I have very little further to suggest. One small thing I would consider is adding that there are no published reports *to your knowledge* in the introduction section of the abstract.

Thank you for the suggestion—we have incorporated this into the text in the introduction section of the abstract.

2. Additionally, as I was reading through this again, I started to wonder how responses may have differed for people for whom the questions were just hypotheticals versus those who were actually eligible for the vaccination and saw people similar to them (in terms of age, etc.) getting the vaccination. Since the covid-19 vaccine rollout in Chad first began in June 2021 and the survey collection ran from August-October 2021, it’s likely that some of the participants would have been eligible for vaccination when they took the survey, while others weren’t, and this might have played some role in levels of vaccine hesitancy (e.g. older people who see others their age getting the vaccine and not experiencing side effects might feel less hesitant than a young pregnant woman who does not know any people who have similar characteristics to her who have gotten a vaccine). Just another thing to consider, but leave it to you whether it seems like a worthwhile additional discussion point.

Thank you for your thoughtful consideration. We agree that this could have played a role in survey responses and have added the following text to the discussion: *“It is also possible due to the close timing of Chad’s COVID-19 vaccine rollout (starting June 2021) and the survey administration (August¬–October 2021) that some of our respondents may have been eligible for vaccination at the time of study participation while others were not, potentially playing a role in their perceptions of vaccination and having an impact on their reports of vaccine acceptance and hesitancy.”*

---

Thank you again for the constructive comments and detailed reviews.
